# Drug Development in Conformational Diseases: A Novel Family of Chemical Chaperones that Bind and Stabilise Several Polymorphic Amyloid Structures

**DOI:** 10.1371/journal.pone.0135292

**Published:** 2015-09-01

**Authors:** Marquiza Sablón-Carrazana, Isaac Fernández, Alberto Bencomo, Reyna Lara-Martínez, Suchitil Rivera-Marrero, Guadalupe Domínguez, Rafaela Pérez-Perera, Luis Felipe Jiménez-García, Nelly F. Altamirano-Bustamante, Massiel Diaz-Delgado, Fernand Vedrenne, Lina Rivillas-Acevedo, Karina Pasten-Hidalgo, María de Lourdes Segura-Valdez, Sergio Islas-Andrade, Eulalia Garrido-Magaña, Alejandro Perera-Pintado, Anaís Prats-Capote, Chryslaine Rodríguez-Tanty, Myriam M. Altamirano-Bustamante

**Affiliations:** 1 Dpto. Neurodiagnóstico, Centro de Neurociencias de Cuba, Cubanacán, Playa, La Habana, Cuba; 2 Unidad de Investigación Médica en Enfermedades Metabólicas, Hospital de Cardiología, Centro Médico Nacional Siglo XXI, Instituto Mexicano del Seguro Social, México D.F., México; 3 Departamento de Microbiología, Escuela Nacional de Ciencias Biológicas, Instituto Politécnico Nacional, México D.F., México; 4 Laboratorio de Nanobiología Celular, Departamento de Biología Celular, Facultad de Ciencias, UNAM, México D.F., México; 5 Instituto de Fisiología Celular, UNAM, México D.F., México; 6 Servicio de Endocrinología, Instituto Nacional de Pediatría, SS, México D.F., México; 7 Cátedra Conacyt, México D.F., México; 8 Servicio de Endocrinología, Hospital de Pediatría, CMNXXI, IMSS, México D. F., México; 9 Centro de Investigaciones Clínicas, La Habana, Cuba; University of Akron, UNITED STATES

## Abstract

The increasing prevalence of conformational diseases, including Alzheimer's disease, type 2 Diabetes Mellitus and Cancer, poses a global challenge at many different levels. It has devastating effects on the sufferers as well as a tremendous economic impact on families and the health system. In this work, we apply a cross-functional approach that combines ideas, concepts and technologies from several disciplines in order to study, *in silico* and *in vitro*, the role of a novel chemical chaperones family (NCHCHF) in processes of protein aggregation in conformational diseases. Given that Serum Albumin (SA) is the most abundant protein in the blood of mammals, and Bovine Serum Albumin (BSA) is an off-the-shelf protein available in most labs around the world, we compared the ligandability of BSA:NCHCHF with the interaction sites in the Human Islet Amyloid Polypeptide (hIAPP):NCHCHF, and in the amyloid pharmacophore fragments (Aβ17–42 and Aβ16–21):NCHCHF. We posit that the merging of this interaction sites is a meta-structure of pharmacophore which allows the development of chaperones that can prevent protein aggregation at various states from: stabilizing the native state to destabilizing oligomeric state and protofilament. Furthermore to stabilize fibrillar structures, thus decreasing the amount of toxic oligomers in solution, as is the case with the NCHCHF. The paper demonstrates how a set of NCHCHF can be used for studying and potentially treating the various physiopathological stages of a conformational disease. For instance, when dealing with an acute phase of cytotoxicity, what is needed is the recruitment of cytotoxic oligomers, thus chaperone F, which accelerates fiber formation, would be very useful; whereas in a chronic stage it is better to have chaperones **A**, **B**, **C**, and **D**, which stabilize the native and fibril structures halting self-catalysis and the creation of cytotoxic oligomers as a consequence of fiber formation. Furthermore, all the chaperones are able to protect and recondition the cerebellar granule cells (CGC) from the cytotoxicity produced by the hIAPP_20–29_ fragment or by a low potassium medium, regardless of their capacity for accelerating or inhibiting *in vitro* formation of fibers. *In vivo* animal experiments are required to study the impact of chemical chaperones in cognitive and metabolic syndromes.

## Introduction

Developing innovative and cost-effective strategies for diagnosing and treating conformational diseases (CDs) is a major challenge of modern biomedical science. Misfolded proteins (more than 40 different kinds) play a central role in the pathophysiology of CDs such as Alzheimer's disease (AD), Huntington's disease (HD), Parkinson's disease (PD), type 2 Diabetes Mellitus (DM2), and Cancer among others. Molecular mechanisms by which an initially innocuous protein adopts an amyloidogenic conformation capable of forming fibers and toxic oligomers, contributing to the onset of CDs is an unanswered question as of yet [1–4]. It is known that sequence-independent amyloid states are formed by elongated fibers with spines built out of β-sheets strands. β-sheets interact with each other by means of a dense hydrogen-bond network, forming a closed dry surface. These structures, known as “steric zippers”, are repeated along the fiber [2,5,6]. In order to reach a minimum in their total free energy, most misfolded proteins, regardless of their structure, can form amyloid aggregates as alternative packing [7–9]. Finding ways to disrupt the formation of amyloid structures, therefore, could lay the foundations for developing novel drugs to prevent, delay the onset, and treat these diseases [10].

Take as example the β amyloid protein (Aβ) whose polymorphic aggregates (toxic Aβ oligomers, protofilaments and fibers) and its circulation or deposition in brain, are the pathological hallmarks of AD. Similarly, recent studies suggest that the aggregation of Human Islet Amyloid Polypeptide (hIAPP), a 37-residue peptide produced in pancreatic beta cells, is a key factor in the development of diabetes [6]. Its fibrillating core, the decapeptide SNFFGAILSS (hIAPP_20–29_), is thus a key point of study. These peptides, however, are costly, and thus inaccessible for large-scale use in some research facilities. In this article we show that Bovine Serum Albumin (BSA), a familiar, ‘off-the-shelf’ protein shares a common meta-structure with IAPP and the amyloid pharmacophore fragments (Aβ_17–42_ and Aβ_16–21_). Systematic comparison suggests that BSA has remarkable potential as a model molecule for further study in the field of pharmaceutics in conformational diseases.

BSA, a protein highly abundant in blood, is composed by 585 amino acids. Under normal conditions, 67% of the BSA’s tertiary structure is in an α-helix conformation [11]. This protein has a globular and rigid structure that can suffer conformational changes depending on the medium’s condition from their native α-helix structure to a β-sheet structure. Although there have been numerous studies investigating the mechanisms of BSA aggregation, the conditions which elicit such conformational changes are still unknown [12]. Battacharya, et al.[12] have found that pH and ionic strength significantly influence the kinetics of aggregation of BSA, while Holm, et al.[13] point out that high temperatures also promote the formation of fibers, indicating that BSA first needs to go into a denatured state in order to aggregate. It has also been proven that various conditions can elicit the formation of structurally different oligomers [7,9,13,14].

As part of the quality control system of proteins, there is a group of molecules known as chaperones. These are proteins that interact with, stabilize, or help other proteins acquire their functionally active conformation, without affecting their final native structure [15]. Several different compounds are classified as chaperones due to their ability to interact with protein [16]. Chaperones have been typified as molecular, chemical, and pharmacological [17–19]. They rescue the functionality of other proteins and promote their correct folding by temporarily binding to partially unfolded intermediaries to prevent their aggregation and interactions [16,20]. It is of great interest, therefore, to study and develop compounds that can bind to a wide variety of polymorphic aggregates, marking them and inhibiting their development. Various studies have proved that there are several proteins, lipids, and small chemical compounds that can inhibit protein aggregation into amyloid structures, acting like chaperones. Worth of mention among these, are Non-Steroid Anti-inflammatory Drugs (NSAIDs), curcumin, heparin, small heat shock proteins (sHsp), inositol derivatives, and many other polycyclic, polyphenolic, and aromatic substances [2,21–25]. Other substances, such as Gemini surfactants, have been studied because of their property of refolding. Some naphthalenes derivatives, such as 2-dialkylamino-6-acylmalononitrile (DDNP, **J**), substituted naphthalenes, and Orange G (**I**) [2], have been likewise successfully bound to amyloid fibers by forming aromatic interactions within the steric zippers’ dry surface, thus disrupting their structure.

**Fig 1:**
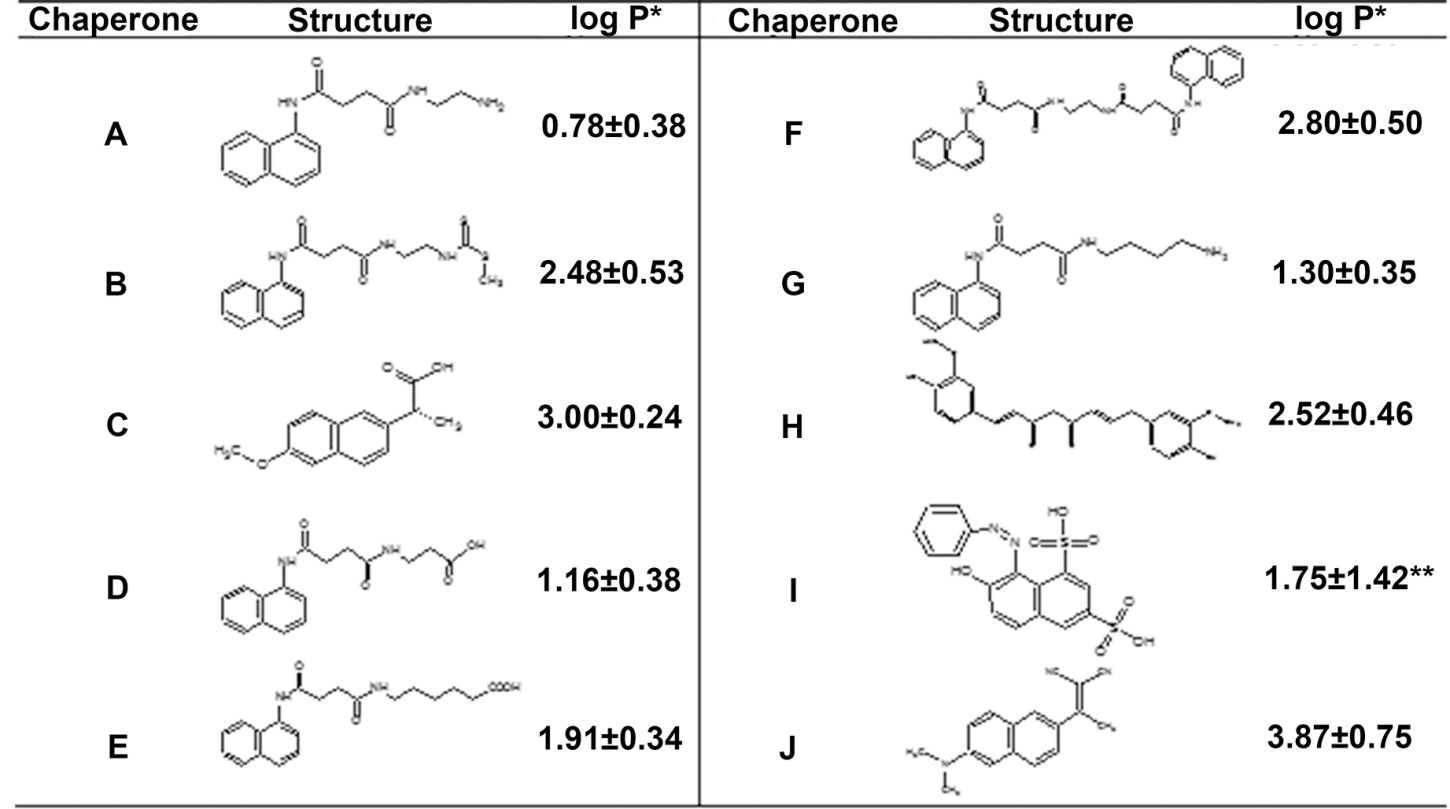
Structures and LogP of the chemical chaperones. *N*-(2-aminoethyl)-*N*'-1-naphthylsuccinamide **A**; methyl (2-{[4-(1-naphthylamino)-4-oxobutanoyl]amino}ethyl) dithiocarbamate **B**; (2*R*)-2-(6-methoxy-2-naphthyl)propanoic acid (*Naproxen*) **C**; *N*-[4-(1-naphthylamino)-4-oxobutanoyl]-β-alanine **D**; 6-{[4-(1-naphthylamino)-4-oxobutanoyl]amino} hexanoic acid **E**; *N* ^3^,*N* ^3^'-ethane-1,2-dyilbis(*N* ^1^-1-naphthylsuccinamide) **F**; *N*-(4-aminobutyl)-*N*'-1-naphthylsuccinamide **G**; (1*E*,6*E*)-1,8-bis(4-hydroxy-3-methoxyphenyl)octa-1,6-diene-3,5-dione (*Curcumine*) **H**; 7-hydroxy-8-[(Z)-phenyldiazenyl]naphthalene-1,3-disulfonic acid (*Orange G*) **I**; (2Z)-3-[6-(dimethylamino)-2-naphthyl]-2-isocyanobut-2-enenitrile (*DDNP*) **J**. *The logP was calculated using *ACD/Log P software (ACD/1-Lab Service*, *Toronto*, *Ontario*, *Canada)*. ** Value estimated *for uncharged compound*.

In this work we apply a cross-functional approach that combines ideas, concepts and technologies from several disciplines to study, *in silico* and *in vitro*, a Novel Chemical Chaperones Family (NCHCHF) The NCHCHF is composed by: N-(2-aminoethyl)-N'-1-naphthylsuccinamide **A**; methyl (2-{[4-(1-naphthylamino)-4-oxobutanoyl]amino}ethyl) dithiocarbamate **B**; N-[4-(1-naphthylamino)-4-oxobutanoyl]-β-alanine **D**; 6-{[4-(1-naphthylamino)-4-oxobutanoyl]amino} hexanoic acid **E**; N3,N3'-ethane-1,2-dyilbis(N1-1-naphthylsuccinamide) **F** and N-(4-aminobutyl)-N'-1-naphthylsuccinamide **G**. Also, the (2R)-2-(6-methoxy-2-naphthyl)propanoic acid (Naproxen) **C**, was used as control, because its anti-amyloid activity reported [26] (Fig 1) [27,28] and their role in protein aggregation in conformational diseases; using BSA and the hIAPP fragment 20–29 (hIAPP_20–29_) considered among the most amyloidogenic of their class. We reveal that some members of NCHCHF have the ability to interact directly with native and/or aggregation-prone species in solution and/or fiber structures to decrease the amount of toxic oligomers in solution. Furthermore they are able to protect and recondition the cerebellar granules cells (CGC) from the citotoxicity produced by hIAPP_20–29_.

## Results

### Molecular Frameworks of Action of Chemical Chaperones

In order to understand the ligand–protein interactions, we used the molecular docking technique to design and test a novel chemical chaperones family using BSA, hIAPP_1–37_, and the amyloid pharmacophore fragments (Aβ_17–42_ and Aβ_16–21_). as target molecules. All these structures were downloaded directly from the PDB: 3VO3, 2L86, 2BEG and 1IYT codes, respectively. Six novel compounds and four references were docked into the 3D structure of BSA using AutoDockVina. The compounds were clustered into four sets, according to the terminal functional group of the amidoalkylic chain and the logP values (calculated by ACD program. *http*:*//www*.*acdlabs*.*com/resources/freeware/chemsketch/logp*), as: 1G (**A** and **G**), 2G (**D** and **E**), 3G (**B** and **F**) and 4G (**C**, **H**, **I** and **J**), the last one being considered as reference. The blind docking simulation protocol points out the most probable binding sites, poses and the energies between the chemical chaperones and the protein.

### BSA Molecular Docking

**Fig 2:**
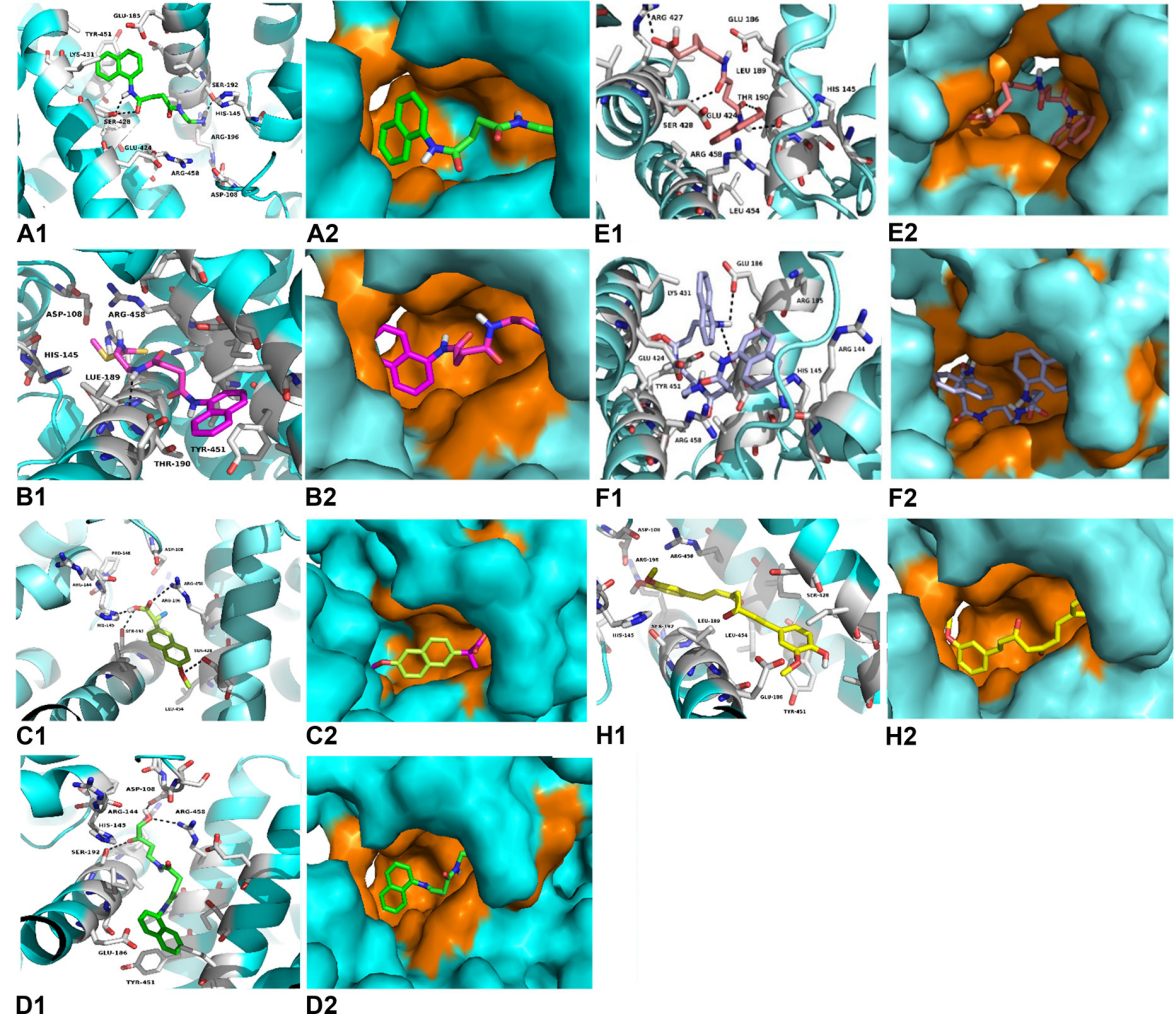
Molecular docked model of chemical chaperones (stick representation), located within the hydrophobic pocket of BSA (cartoon and sphere views). At 5Å distance, the amino acid residues surrounding chaperones (**A**, **B**, **C**, **D**, **E**, **F**, **I** and **H**) are represented in orange. The H-bonding interaction between chaperones and amino acid residues of BSA, are shown as a dotted line.

Observations of the 3D structure of crystalline albumin have shown that BSA is constituted by three homologous domains (I, II, III): I (residues 1–183), II (184–276) and III (377–583), each one containing two subdomains (A or B). Two hydrophobic cavities are located within the sub-domains IIA and IIIA, which are considered drug-binding sites. Its secondary structure is dominated by an α-helix. The binding sites of all the chaperones tested with BSA were in the similar vicinity. The main regions of ligand binding to BSA were located in hydrophobic cavities of the subdomains IIA and IIIA, known as drug’s pocket. This site is a large hydrophobic cavity, which accommodates chaperone molecules. Examples of the location of these sites in the 3D structure of BSA are shown in Fig 2.

**Fig 3:**
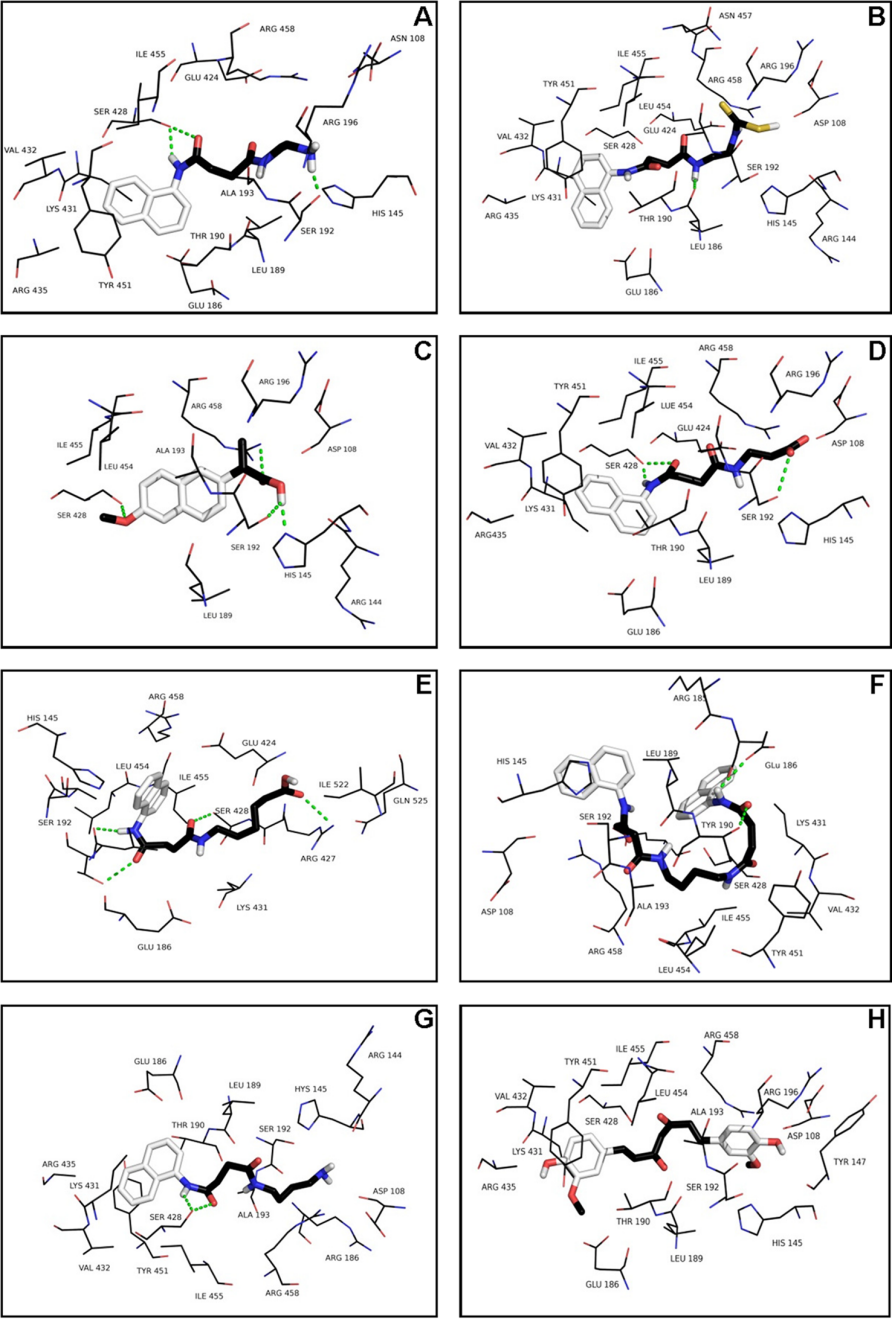
Visualization of binding site of chaperones (A, B, C, D, E, F, G and H) into IIA and IIIA regions of BSA, within 5Å distance.

The conformer with the lowest binding energy was calculated from 10 different conformations for each docking simulation, at 5 Å distance between chaperones and BSA (S1 Table). Finally, it was found that the chaperone-BSA binding site was located into the domain IIA and IIIA of the protein, which corresponds to the lowest free energy values calculated. According to the analysis of the calculated free energy, the binding process of BSA with chaperones is spontaneous (ΔG<0). The common amino acid residues lining this binding site were: His145, Glu186, Leu189, Thr190, Ala193, Arg196, Glu424, Ser428, Lys431, Val432, Arg435, Tyr451, Ile455, Arg458 and Asp107, Ser109, Leu189, Thr190, Ala193, Arg196, Glu424, Ser428 Lys431, Val432, Arg435, Tyr451, Ile455, Arg458, for the 1G and 2G groups respectively. The compounds of the 3G group interact with the following common amino acid residues: Asp108, Glu186, Leu189, Thr190, Ser192, Ala193, Arg196, Glu424, Ser428, Lys431, Val432, Arg435, Tyr451, Leu454, Ile455, and Arg458. Similarly, the binding amino acid residues for **C** and curcumin (**H**) are Glu186, Leu189, Thr190, Ser192, Ala193, Arg196, Glu424, Ser428, Lys431, Val432, Arg435, Tyr451, Ile455, and Arg458. In 85% of calculations, the interaction zone was comprised between amino acids 145 to 458, specifically with the residues His145, Glu186, Leu189, Thr190, Ser192, Ala193, Arg196, Glu424, Ser428, Lys431, Val432, Arg435, Tyr451, Ile455, and Arg458. In general, these molecules bind to the sites via hydrophobic and Van der Waals forces. In some cases, hydrogen-bonding interactions have been observed. (Fig 3).

Taking chaperones **A**, **B**, **D** and **H** as representative examples of the different groups (1G, 2G, 3G and 4G), the docking results reveal that the chaperones bind to the sites IIA and IIIA of BSA, mainly via hydrophobic interactions with the Leu189, Ala193, Val432, and Ile455 residues, as well as by Van der Waals forces. The interactions π∙∙∙π were located between the naphthyl ring of the chaperones and the phenyl ring of the Tyr451. In the case of the chaperones **A** and **D** there was hydrogen bonding with two residues of Ser (198 and 428) of the BSA. In the case of chaperone **B**, this interaction was found with Leu189. Otherwise **H** did not expose the hydrogen bonding interaction (Fig 3). The binding process of BSA with chaperones is spontaneous (ΔG<0). The interaction forces are mainly hydrophobic and hydrogen bonding.

### Amyloid Pharmacophore Molecular Docking

Eisenberg et al.[2] demonstrated that amyloid pharmacophores, offering cavities along β-sheets, are similar to those observed binding of amyloid markers to β-sheets in non-fibrillar structures and the oligomers and fibers that are inhibited by similar compounds, including **H**. The amyloid model is used as target for docking simulations in order not only to understand the aggregation process, but also to find compounds that could prevent the formation of β-structure. Also, three crystal structures of small molecules bound to amyloid-like segments of *tau* and amyloid-β (**I**, **H** and **J**) were reported. In this work, they described the molecular framework of small-molecule binding, within cylindrical cavities running along the spines of the fibers. The segment selected for Eisenberg (KLVFFA, residues 16–21) corresponding to Aβ_1–42_ has also been reported by other authors as an amyloidogenic region [29–31]. This peptide segment contains two Phe residues and a charged amino acid (Lys), involved in the formation of aromatic interactions and salt bridges, which allow the formation of the α/β-structure.

**Fig 4:**
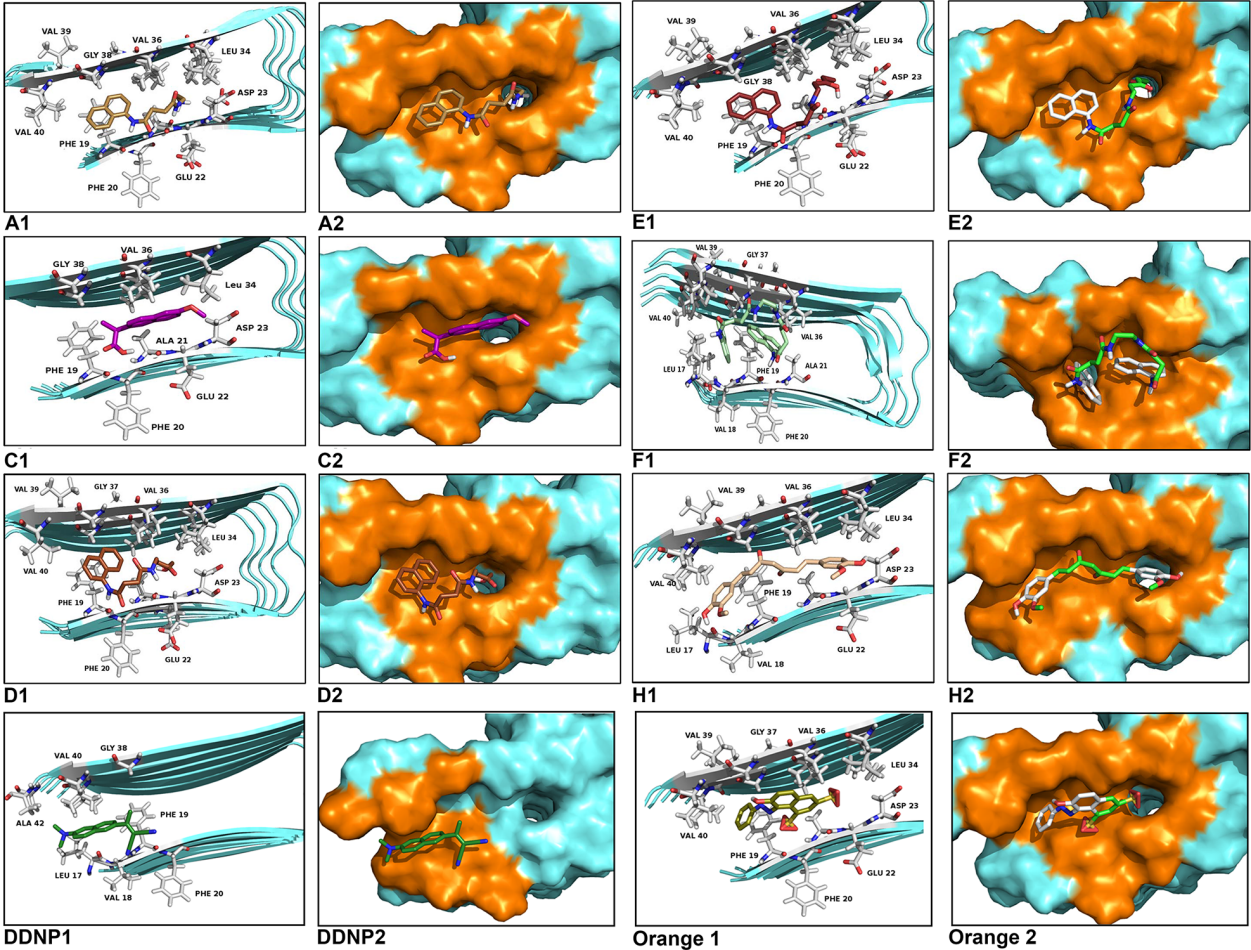
Molecular docked model of chemical chaperones (stick representation), located in the amino acids of Aβ_17–42_ segment (cartoon and sphere views). At 5Å distance, the amino acid residues surrounding chaperonins (**A**, **B**, **C**, **D**, **E**, **F**, **H**, **I** and **J**) are represented in orange. The hydrogen bonding interaction between chaperones and amino acid residues of Aβ_17–42_ segment are shown as a dotted line.

Pharmacophore-based virtual screening is a widely used strategy for drug design based on the identification of novel hits. As a part of on-going effort to identify novel chemical chaperones for potential drug development, we used the pharmacophoric features (the atomic structures of the adhesive segments of amyloid fibers, termed steric zippers) of Aβ_17–42_ in its fibril model, for docking studies that reveal the interaction between them and our set of chemical chaperones. Fig 4 shows the interaction sites between the chaperones and the amino acids of the Aβ_17–42_ segment, represented as a 3D structure. All the simulations demonstrate that the chaperones interact with two regions, mainly with the amino acid residues: Leu17, Phe19, Phe20, Ala21 and Met36, Val37, Gly38, Gly39, Val40. These amino acids are involved in the so called steric zipper of the Aβ_17–42_ segment, which several studies report as key in the formation of fibrils [2]^,^[29–31]. The docking results reveal that the chaperones bind to the Aβ_17–42_ region, essentially via hydrophobic interactions and by Van der Waals forces, mainly with the above-mentioned residues. The interactions π∙∙∙π were located between the naphthyl ring of the chaperones and the phenyl ring of the Phe19 and Phe20. Also, the weak interactions by salt bridge between Asp23 and the chaperone **F**, **H**, **I** and **J** were observed. Our docking results are in good agreement with the ones reported by Eisenberg et al [2].

**Fig 5:**
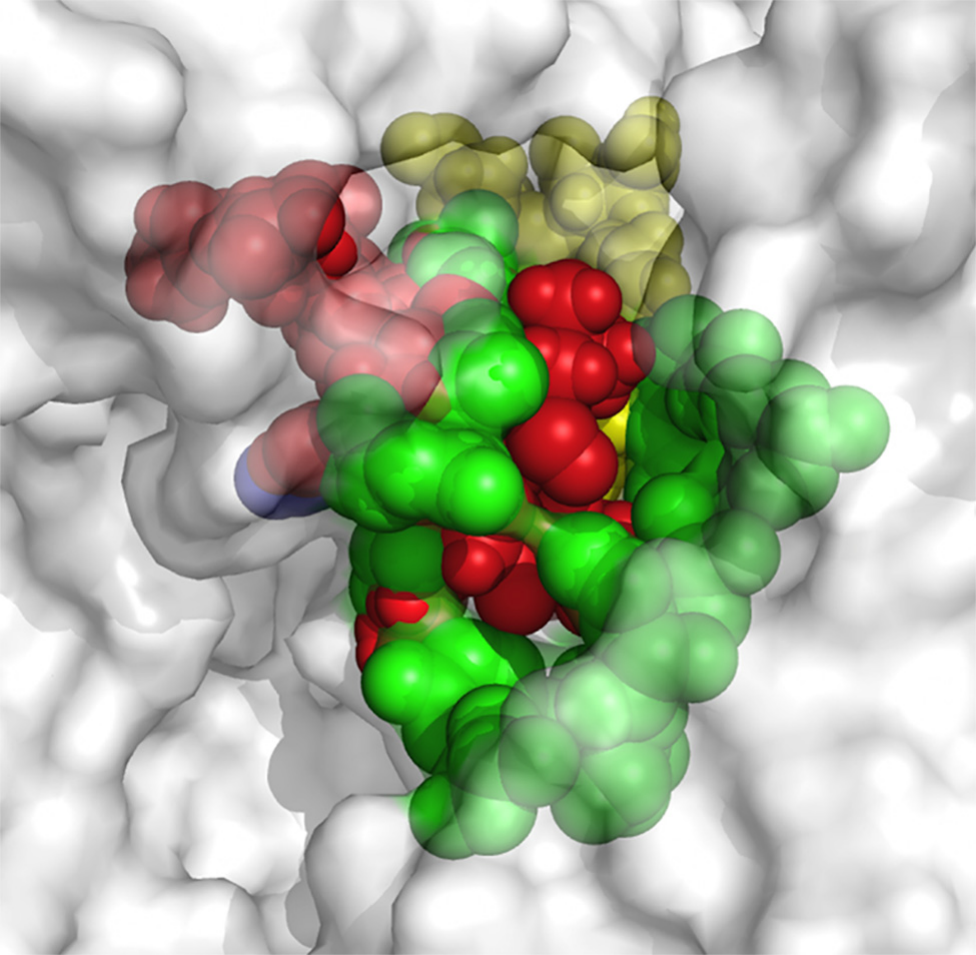
Meta-structure of pharmacophore. A structural alignment using Pymol, whereby the interaction zones between β-amiloyd_17–42_ (Aβ_17–42_), IAPP and Eisenberg’s pharmacophore molecules and the chaperons were set in position with the interaction zone between BSA and the chaperons. BSA is shown in white at 70% transparency, the interaction zone between the Aβ_17–42_ and the chaperons is shown in red, the one corresponding to the Eisenberg pharmacophore in blue, with IAPP in yellow and with BSA in green.

Protein-ligand docking is the most intuitive and powerful tool for structure-based drug discovery. Using protein-structure similarity clustering, as described by Waldmann and co-workers [32–36], we identified the conservation of structural motifs in the binding region of four target proteins (BSA, Aβ_17–42_, hIAPP_1–37_ and Aβ_16–21_, the two last data no shown) with the chaperones as ligands. First, the interaction zones of each of them were identified. So, structural alignment using Pymol was performed between each building blocks and root mean square deviation values (RMSD ‹ 3Å) were obtained for each case. These low RMSD values indicated the 3D structural similarity of the interaction sites between all proteins simulated (Fig 5). This means that the conservation of the structural motifs, as the ligand recognizing area, allows us to target these regions, which can well be called a “meta-structure” viewed as an intricate network of interacting residues in a topological space, according to Konrat et. Co-workers [35,36]. In sum, this strategy follows the principle that identifying meta-structure similarities in ligand interaction sites is a reliable method for discovery chemical scaffolding which serve as guides toward the development of new drugs. Moreover, it is possible to speak of a “meta-pharmacophore” that allows the development of compounds that prevent aggregation at different stages; that is to say, from stabilizing the native state to destabilizing the oligomeric state and protofilament formation, which is the case of interest to this paper.

### Influence of Potential Drugs on Fibril Formation Monitored by Thioflavin T Fluorescence

**Fig 6:**
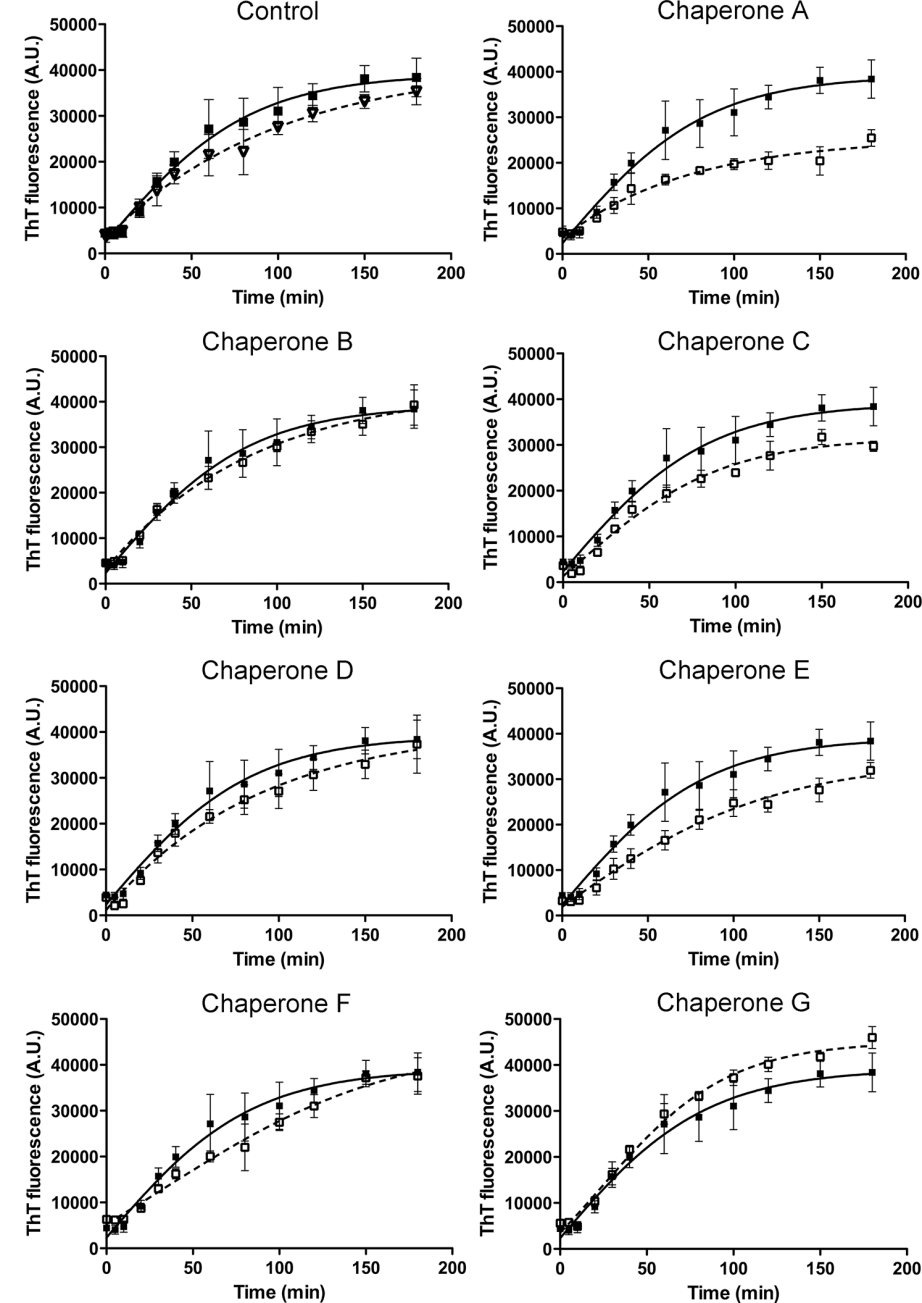
A. ThT fluorescence kinetics during amyloid fibrillation of BSA. Experiments were carried out at 65°C in glycine buffer (pH = 3; 0.05M, NaCl 100 mM), in presence or absence of the selected chaperones at molar relation 1:1 of BSA: Chap. (ThT: 24 μM). Time dependent changes in ThT intensity was fitted by exponential function (solid line). In all cases, the adjusted R-square values were greater than 0.9. The experiments were carried out in triplicate. The control BSA alone (black square) and BSA plus DMSO (white triangles). All the chaperones (empty squares) were compared with BSA plus DMSO (black squares). B: Normalized maximum values of the ThT fluorescence intensity of BSA fibrilations in presence or absence of chaperones (**A**, **B**, **C**, **D**, **E**, **F** and **G**).

The Thioflavin T (ThT) fluorescence is widely used to study the kinetics of amyloid fibril formation in different proteins [13]. According to the studies consulted, the fibril formation mechanism of BSA is distinguished from others on account of its mechanism of aggregation, which is composed of two states: a logarithmic phase of rapid growth, corresponding to fibril elongation, and a plateau phase of stationary growth, during which there may be continuing morphological changes that can be fitted into a single exponential decay without any lag phase. The ThT fluorescent assay was used to monitor the fibril formation of BSA in the presence or absence of the novel family of chemical chaperones. NCHCHF were added to the ThT assay, in a molar ratio of 1:1 BSA: chaperone. Fig 6A shows *ex situ* measurements of ThT emission intensity with and without chaperones. Triplicate aggregation kinetics were developed at different times at 65°C. The samples were cooled in order to stop the aggregation process as described by Vetry et al. [9] BSA aggregation was significantly inhibited with increasing doses of chemical chaperones **A**, **C**, **D** and **E**, with A IC50 of 6.34 μmol/L, 9.21 μmol/L, 6.37 μmol/L and 5.38 μmol/L, respectively. Although chaperone **F** (40 μmol/L) showed the highest IC50 value, this compound delayed the BSA aggregation and also diminished the total amount of fiber.

At a concentration of 100 μmol/L (BSA: chaperone ratio of 1:1) chaperones **A**, **C, D, B** and **E** had a strong inhibitory effect on BSA fibrillation and showed a decrease in ThT fluorescence values between 41% and 14% with respect to BSA alone. At the same concentrations, the chaperones **F** and **G** increased BSA fibrillation and had ThT fluorescence values between 22% and 9% greater than BSA alone (Fig 6B).

**Fig 7:**
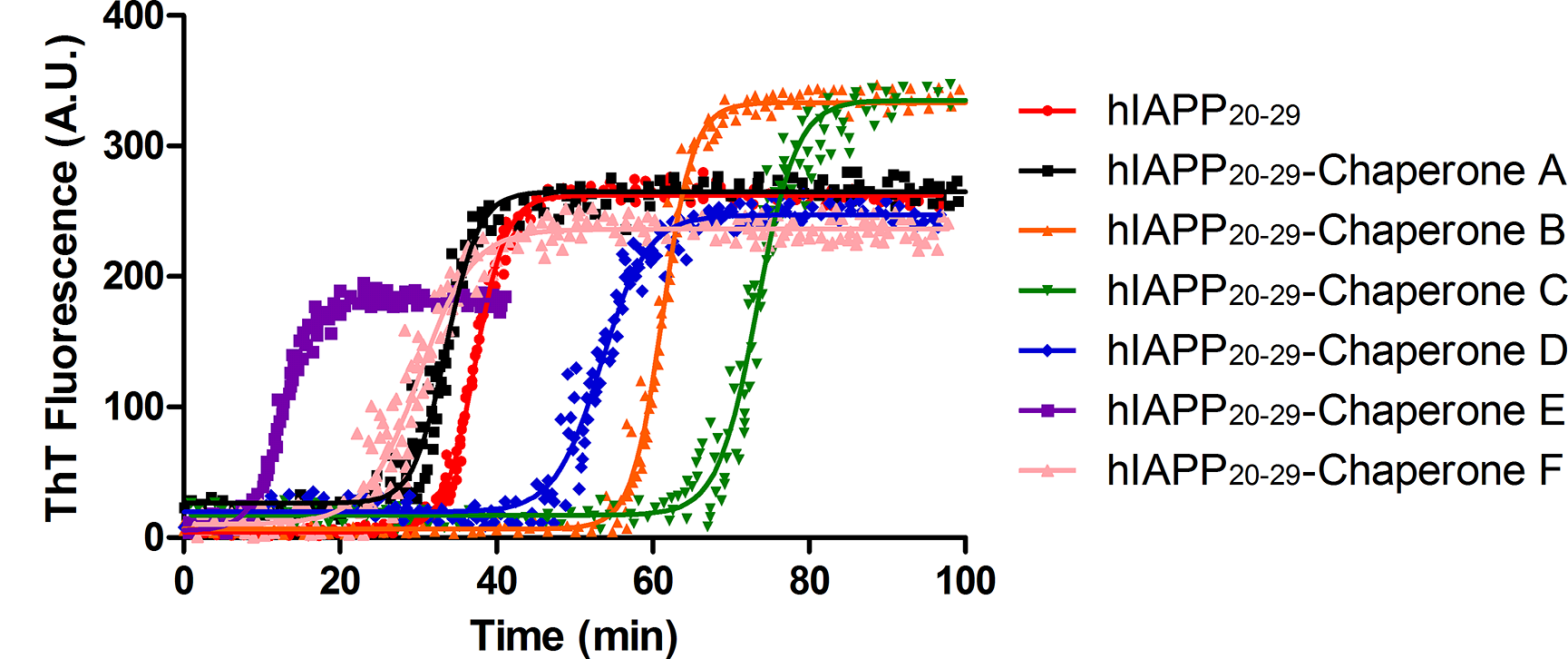
ThT fluorescence kinetics during amyloid fibrillation of IAPP_20–29_ (100 μmol/L). Experiments were carried out at 25°C in PBS buffer (pH 7.4; 10 mmol/L; NaCl 100 mmol/L), in presence or absence of the selected chaperones at molar relation 1:1 of IAPP_20–29_: Chap. (ThT: 24 μM). Time dependent changes in ThT intensity was fitted by sigmoidal function (solid line). The experiments were carried out in triplicate. t_lag_ of fibril formation of the tested chaperones * p < 0,05 show significant differences with regard to control.

On the other hand, experimental studies have shown that hIAPP_20–29_ fragment is capable of forming fibrils *in vitro*. The ThT fluorescence kinetics of hIAPP_20–29_ fragment with or without chemical chaperones are presented in Fig 7. The experiments were performed at 25°C in 100 mmol/L phosphate buffer, at pH 7.4, containing 100 mmol/L NaCl with continuous stirring. The results indicate that chaperones **B**, **C** and **D** can bind to native hIAPP_20–29_ fragment and delay fibril formation (t_lag_ of 48.2 ± 9.4, 69.9 ± 0.9 and 47.8 ± 1.6, respectively) with a t_lag_ higher and significantly different (p < 0.05) than the control (t_lag_ of 31.9 ± 3.2). However, chaperones **E** and **F** accelerate the fibril formation (t_lag_ of 9.7 ± 0.2 and 25.6 ± 0.6 respectively), whereas **A** does not present any modulating effect with respect to control (t_lag_ of 33.6 ± 1.3) (Fig 7).

### The Effects of Potential Drugs on Aggregation Monitored by Circular Dichroism Spectroscopy

The conformational differences of the amyloid-like structure of BSA at 70°C in absence and presence of a NCHCHF were investigated by Circular Dichroism (CD) spectroscopy. The far-UV CD spectra of BSA incubated at room temperature shows minima at 222 and 209 nm, characteristic of a helical structure [12]. After incubating the sample at 70°C for 240 minutes, CD spectra showed changes in protein structure composition obtained from CD analysis. Table 1 shows the secondary structure variation on BSA samples at the end (240 min) of incubation at 70°C in absence and presence of **A**, **B**, **C**, **D**, **E** and **F**. We observed the differences of secondary structure composition (expressed in %) at zero and 240 minutes of incubation. The negative signs stand for a decrease in the corresponding structure, as described by Juárez [7,14]. Within the NCHCHF analyzed here, the content of α-helical structure is negative and larger for BSA alone in comparison to chaperones (**F**>**D**>**B**>**C**>**A**>**E**) (see Table 1). There was a loss in α-helix structure and the formation of both β-sheet and unordered conformation. The amyloid-like fibrils were found to increase β-sheet content in the BSA alone more than in the presence of chaperones (**F**>**D**>**E**>**B**>**A**>**C**) (Table 1). It is important to note that without chaperones, the BSA loses its helical shape, which suggests that the former stabilize the α-helix structure and diminish aggregation.

**Table 1:** Values of molar ellipticity differences of the BSA fibrilogenesis assays at 70°C, in absence and presence of chaperones A, D, E, B, F and C, calculated from the CD spectra.

	Δ(Θ_240min_- Θ_0min_) (%) *		
	Δ α-helix	Δ β-sheet	Δ others
BSA	-24.2	15.3	7.0
BSA:**A** (1:1)	-20.5	12.9	6.6
BSA:**B** (1:1)	-22.2	13.7	6.2
BSA:**C** (1:1)	-16.5	8.0	5.2
BSA:**D** (1:1)	-19.8	9.0	5.4
BSA:**E** (1:1)	-17.5	7.2	5.3
BSA:**F** (1:1)	-15.1	9.2	5.0

*Differences of molar ellipticity at 240 and zero min. of incubation time.

### Electron and Atomic Force Microscopy Revealed the Effects of Potential Drugs in Fibril Formation

**Fig 8:**
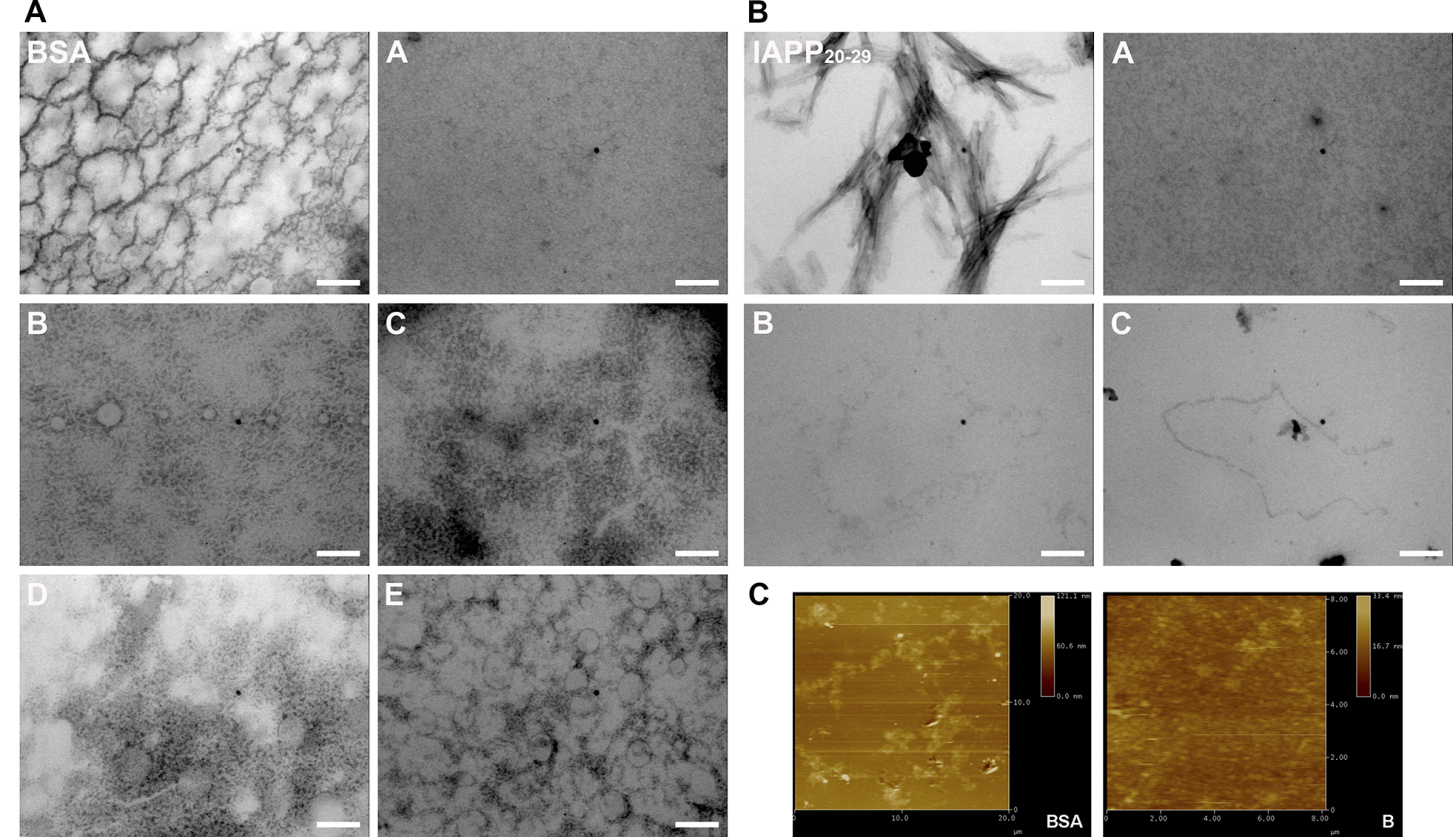
A. Transmission Electron Microscopy (TEM) micrographs of BSA fibrilogenesis process (75 μmol/L), with and without N-(2-aminoethyl)-N'-1-naphthylsuccinamide A; methyl (2-{[4-(1-naphthylamino)-4-oxobutanoyl]amino}ethyl) dithiocarbamate B; (2R)-2-(6-methoxy-2-naphthyl)propanoic acid (Naproxen) C; N-[4-(1-naphthylamino)-4-oxobutanoyl]-β-alanine D and 6-{[4-(1-naphthylamino)-4-oxobutanoyl]amino} hexanoic acid E, at 70°C. B: Transmission Electron Microscopy (TEM) micrographs of hIAPP_20–29_ fibrilogenesis process with and without N-(2-aminoethyl)-N'-1-naphthylsuccinamide A; methyl (2-{[4-(1-naphthylamino)-4-oxobutanoyl] amino} ethyl) dithiocarbamate B and (2R)-2-(6-methoxy-2-naphthyl)propanoic acid (Naproxen) C; at 25°C. Molar ratio hIAPP_20–29_: chaperones was 1:1. C: Atomic Force Microscopy (AFM) micrographs of BSA fibrilogenesis process (75 μmol/L), with and without B, at 70°C. Molar ratio BSA:chaperones was 1:1.

The morphology of the BSA and hIAPP_20–29_ aggregates formed with and without chemical chaperones was examined under transmission electron and atomic force microscopies (TEM and AFM, respectively), in order to check the evolution of fibril formation (Fig 8). Fig 8A shows images of BSA fibrillation process at the end of the incubation time (240 min at 70°C). In the first micrograph of BSA alone, the presence of long and curly fibers is observed. In contrast, at room temperature neither fibrils nor other types of aggregates are detected (data not shown), in agreement with previous reported work [12,13]. When the incubation of BSA was carried out in the presence of chaperones it was not possible to distinguish fibers but several amorphous structures were detected. This result was also confirmed by AFM, which provided micrographs at the end of the incubation time of BSA aggregation that show non-fibrillar structures in presence of chaperone **B**, as can be observed in Fig 8C (BSA alone and BSA:**B** (1:1)).

**Fig 9:**
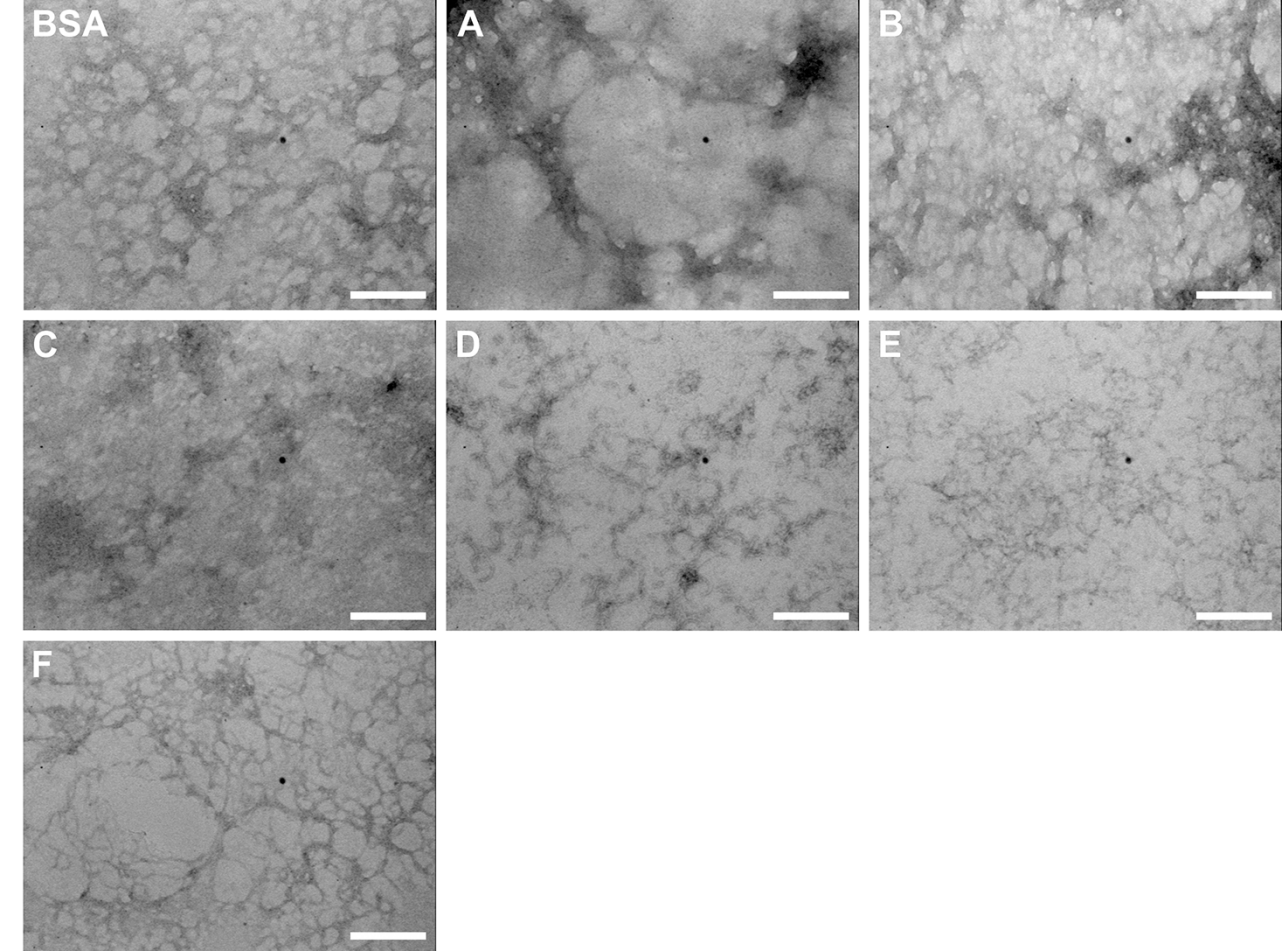
Transmission Electron Microscope (TEM) images of the BSA fibril formed with or without the chaperones (A, B, C, D, E, F and G) at a molar relation of BSA: chaperones 1:1, in buffer Tris (pH = 7.4, 20 mM) and 75°C. Scale bar 200 nm.

A similar modulating effect on the fibril formation of hIAPP_20–29_ was observed in presence of chaperones **A**, **B** and **C**, at a molar ratio of 1:1, at 25°C (Fig 8B). According to TEM micrographs, fibers are evidently formed in hIAPP_20–29_ alone, but in presence of chaperones **A**, **B** and **C** these fibrillar structures were not observed. When BSA was pre-aggregated for 24 h at 75°C before incubation with chemical chaperones **A**, **B**, **C**, **D**, and **E** at 1:1 molar ratio, all of them were capable of disaggregating mature fibrils of BSA (Fig 9).

### Cytoprotective Effect of the Chaperones Family on Cerebellar Granule Cells

**Fig 10:**
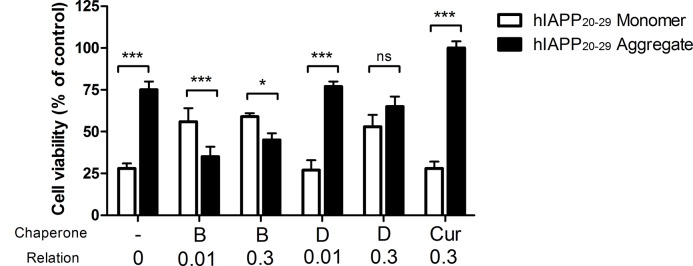
Cell Viability of CGC exposed to hIAPP_20–29_, in monomeric and aggregated form, through MTT assay, with and without N-[4-(1-naphthylamino)-4-oxobutanoyl]-β-alanine D; methyl (2-{[4-(1-naphthylamino)-4-oxobutanoyl]amino}ethyl) dithiocarbamate B and H used as reference; at different molar ratio hIAPP_20–29_:chaperones. All the experiments have the same concentration of hIAPP_20–29_

Cytoprotective properties of chaperones **B** and **D** were evaluated in cerebellar granule cells (CGC) after exposal to hIAPP_20–29_, a toxic protein (in monomeric and aggregated form). This was done by means of viability and apoptosis cellular assays. In all assays, **H** was used as reference due to its reported cytoprotective effect [37]. In Fig 10, the estimation results of cell viability by means of MTT assay is observed. The data was analyzed with statistical test-way ANOVA, followed by a least significant difference (LSD) posteriori test. The results indicate that there are significant differences between the cytotoxic effect of monomeric and aggregated hIAPP_20–29_ forms (p < 0.05). This protein fragment in its monomeric form is nearly three times more cytotoxic to the CGC than in its aggregated form.

Moreover, when cells were exposed to hIAPP_20–29_ in monomeric form and to the chaperones of interest, the viability results were twice higher for the chaperone **B** in the two molar ratios tested, being significantly different from the rest of the other compounds tested (p <0.05). Also, the CGC viability in presence of chaperone **D** was almost twice as high for a molar ratio of 1:0.3 (p <0.05). In contrast, **H**, used as reference, did not show any cytoprotective effect.

**Fig 11:**
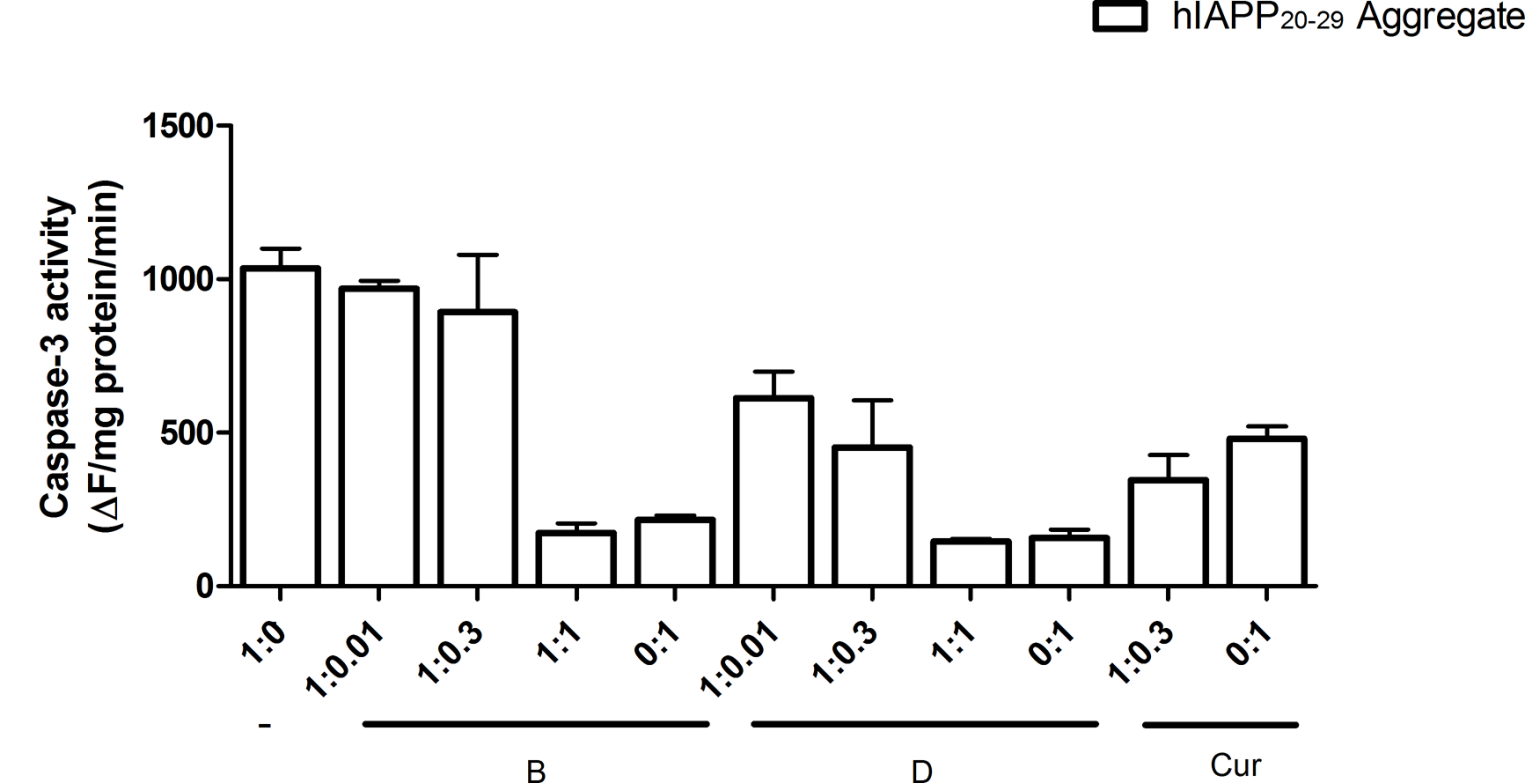
Cell Apoptosis of CGC exposed to hIAPP_20–29_, in monomeric and aggregated form, with and without N-[4-(1-naphthylamino)-4-oxobutanoyl]-β-alanine D; methyl (2-{[4-(1-naphthylamino)-4-oxobutanoyl]amino}ethyl) dithiocarbamate B and H used as reference; at different molar ratio hIAPP_20–29_:chaperones. In all assays caspase-3 levels were measured by immunofluorescence.

In the case of the aggregated form of hIAPP_20–29_ in presence of chaperone **D**, the CGC viability did not differ significantly from cells exposed to hIAPP_20–29_ (p >0.05). As expected, **H** showed a marked cytoprotective effect that differed statistically from chaperones **B** and **D** (p <0.05). Fig 11 shows the results of the cell apoptosis assays, in which the level of caspase-3 was measured by an immunofluorescence assay. The caspase-3 levels of CGC exposed to monomeric or aggregated hIAPP_20–29_ were not significantly different between them (p >0.05) and they were the highest apoptotic levels observed. Contrastingly, in presence of chaperones, the caspase-3 levels of CGC exposed to hIAPP_20–29_ diminished (p <0.05).

When the cells were exposed to the monomeric form of hIAPP_20–29_ in presence of chaperone **B** (molar ratio hIAPP_20–29_:**B**, 1: 0.01 and 1: 0.3), the caspase-3 levels were similar to those obtained with the protein in its monomeric form (p >0.05). However, in the case of chaperone **D**, the caspase-3 levels were lower, but without reaching the levels obtained for **H** in a molar ratio of 1:0.3. Lower caspase-3 levels were achieved for a molar ratio hIAPP_20–29_: chaperone 1:1 for both chaperones (p <0.05). Thus, a six to seven times higher survival average of CGC was observed in the presence of chaperones **B** as for **D**, with regards to exposition to hIAPP_20–29_ alone.

### Reconditioning Activity of Chaperones B, C and D on CGC

In this study the effect of chaperones **B**, **C** and **D** on the survival of CGC, exposed to a pro-apoptotic stimulus, in this case the reduction of potassium concentration (from 25 mmol/L to 5 mmol/L) was evaluated. In this experiment the CGC were exposed individually to these chaperones at different times: at initial time (Group I) and after 4 h of stimulus induction (Group II). Two controls were also used at 25 mmol/L (K25) and 5 mmol/L (K5).

**Fig 12:**
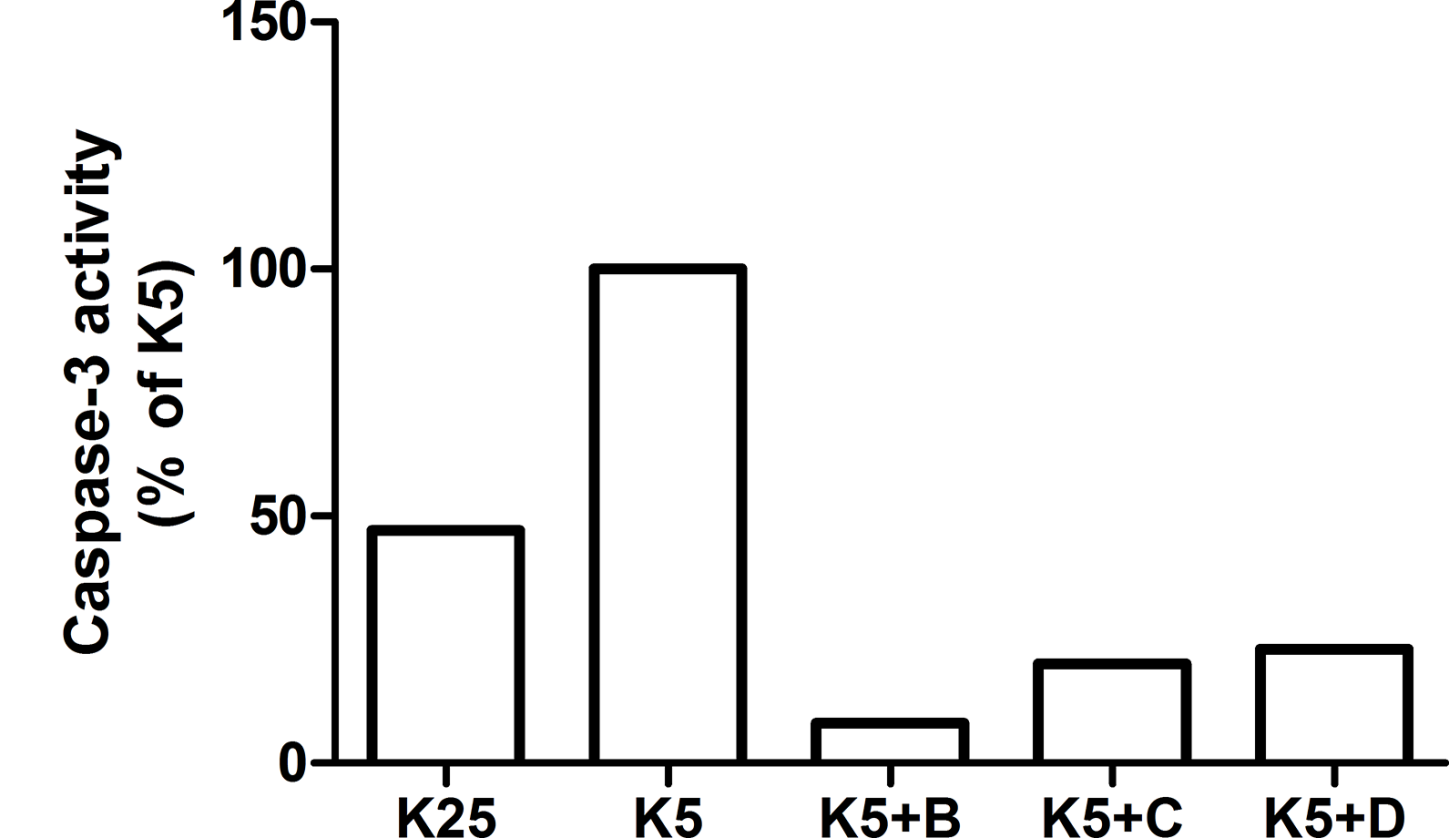
Cell Apoptosis of CGC exposed to pro-apoptotic stimulus as low potassium concentration (K5) in the presence of N-[4-(1-naphthylamino)-4-oxobutanoyl]-β-alanine D; methyl (2-{[4-(1-naphthylamino)-4-oxobutanoyl] amino}ethyl) dithiocarbamate B and C used as reference. In all assays caspase-3 levels were measured by inmunofluorescence.

Group I (Fig 12), treated with chaperones **B**, **C** and **D**, showed a considerable reduction in the caspase-3 levels, with regard to control K5. This reduction was of 92%, 80% and 78% for the samples treated with **B**, **C** and **D**, respectively. With regard to control K25, it was observed that chaperone **B** reduced the caspase-3 level in 84%, while **C** and **D** did so in a 57% and a 52%, respectively.

**Fig 13:**
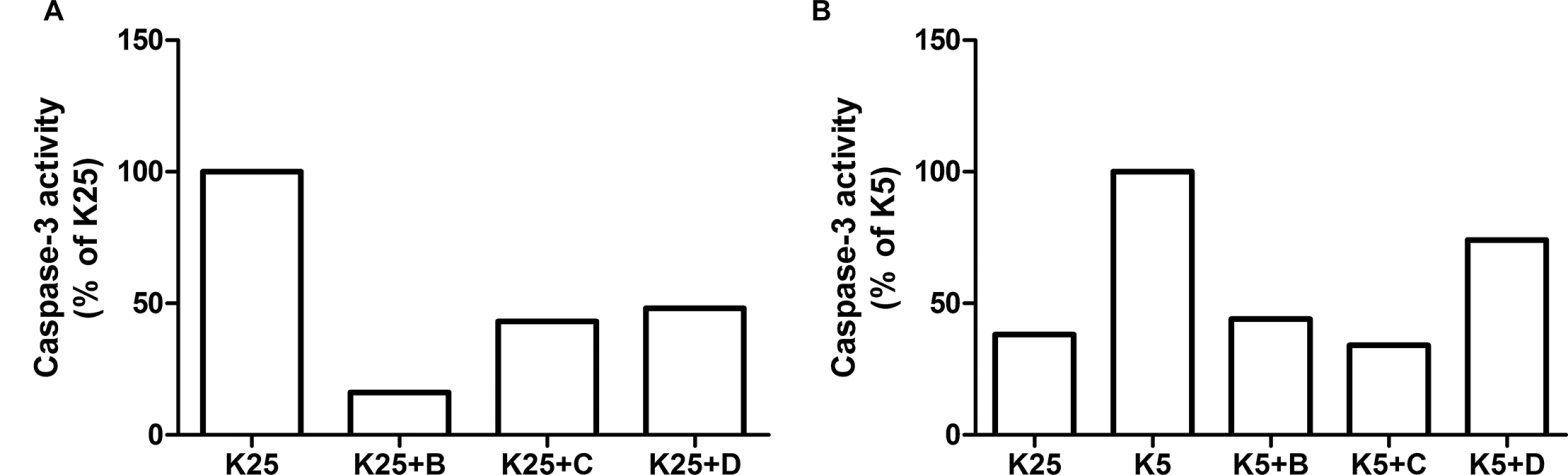
Cell Apoptosis of CGC exposed to pro-apoptotic stimulus as low potassium concentration (K5) during 4 h and later, the chaperones N-[4-(1-naphthylamino)-4-oxobutanoyl]-β-alanine D; methyl (2-{[4-(1-naphthylamino)-4-oxobutanoyl]amino}ethyl) dithiocarbamate B and C (reference), were inoculated. In all assays caspase-3 levels were measured by inmunofluorescence.

In group II (Fig 13), treated with chaperones **B**, **C** and **D**, the caspase-3 levels were lower in a 56%, 66% and 26%, respectively, relative to the control group K5, which is associated with increased cell survival. The CGC with **B** and **C** show values that are very close to those of control test K25, whereas the cells exposed to **D** had an activity that is 94% higher. These results indicate that the chaperones tested were able to reverse the apoptosis induced in CGC through a low potassium environment.

## Discussion

In this article, we show how a novel chemical chaperones family (NCHCHF) can regulate fibrillogenesis of target proteins, as they interact with both the native and fibrillar structures. Chaperones **A**, **B**, **C** and **D** arrest the aggregation of BSA and hIAPP_20–29_ and prevent the formation of potentially cytotoxic species. Conversely, chaperones **E**, **F** and **G** caused the t_1/2_ to be prolonged in the BSA aggregation. It is worth noting that in the case of hIAPP_20–29_, chaperones **E** and **F** accelerate the process of fibril formation. One possible explanation for this phenomenon is related to the fact that chaperones stabilize the structure of the fiber, shifting the equilibrium towards fiber formation and to halt self-catalysis and the creation of cytotoxic oligomers as a consequence of fiber formation. The differential effects of chemical chaperones on the fibril formation of BSA and hIAPP_20–29_ are similar to the results reported for Blancas y coworkers on the t50 value of fibril formation kinetics of AL proteins [38].

That the ThT intensity decreases in the presence of chaperones, indicates a decrease in β-rich amyloid-like aggregates, which is corroborated when exposed to far-UV CD (Fig 6 and Table 1). The TEM experiments show that without chaperones BSA can form long and curled fibers, but not thick ones. This is consistent with a number of studies [7,9,13]. When BSA at 70°C is treated with chaperones **A**, **B**, **C**, **D**, **E**, **F** and **G**, smaller fibers, amorphous aggregates, and ring shaped oligomers are formed. Holm, et al.[13] have found that the structure of the fibrillation intermediaries is highly dependent on the protein-solvent interactions, and the conditions of the medium. Therefore, we assume that the structural variation found in the treated samples is due to a change in the type of interactions that the proteins establish with the solvent and chaperones. Chaperones **A**, **B**, **C** and **D** proved to be more effective than chaperones **E**, **F** and **G** in the disaggregation processes of BSA [22,39].

The molecular simulation indicates that the interaction between BSA and the chaperones is mainly of two kinds: hydrophobic and hydrogen bonding. A similar behaviour has been previously reported for other small molecules [22,40–45]. These interactions, furthermore, play a crucial role in the binding process of chaperones with BSA, which is consistent with our experimental results. The bioinformatic results and the kinetics experimental results are correlated (Figs 3 and 6) since chaperones decrease the fibril concentration and its formation rate. This process evidently occurs on account of the interaction of these compounds with protein thus modulating the transformation from α-helix to β-amyloids-like structure. In accordance to experimental results, we demonstrated that this set of chemical chaperones can influence protein aggregation and fibril formation, as well as protecting cerebellar granule cells from apoptosis upon exposure to hIAPP_20–29_. A possible explanation of these results, based on virtual screening studies, is that these chaperones interact with the amino acids of the so-called amyloid-like steric zipper zone (through Van der Waals forces, interactions π∙∙∙π and H-bond), which are the binding sites of the proteins superposed in the meta-structure, defined as meta-pharmacophore by us (Fig 5). Although the steric zippers cannot have all the features of the full amyloid target proteins, they share many properties with them, thus shedding light upon the functional attributes of specific and broad-spectrum amyloid binders. These findings agree with and build on the work of Eisenberg et al.[2] by arguing that there might be a universal pharmacophore in proteins, which forms a common meta-structure in which chemical chaperones can be bound even if it have different effects. From this follows that, being akin to hIAPP in important respects, BSA can serve as an affordable and accessible model protein that can be used when evaluating new therapeutic agents for conformational diseases.

The development of new compounds with anti-amyloid activity is of great interest for neuroscientists and diabetes researchers. While all of the chaperones tested showed a modulating effect of BSA and hIAPP_20–29_ aggregation, the mechanisms by which these bind to the protein, either to arrest, accelerate the fibrillation process, or to disaggregate fibers is shown in Figs 6, 8 and 9. Further research using *in vivo* studies could be of great use for determining the mechanism of action of these chaperones, thus laying a stronger foundation from whence to develop other compounds.

### Mechanistic Insights

While several studies focus on finding novel therapeutic agents to fight conformational diseases, they are still only moderately successful [22–24]. Recent work on the atomic structure of a segment of the steric zipper of the amyloid-beta (Aβ) pointed out at new compounds with potential as therapeutic agents, which diminish the cytotoxicity of the Aβ but do not reduce the formation of fibers [46].

**Fig 14:**
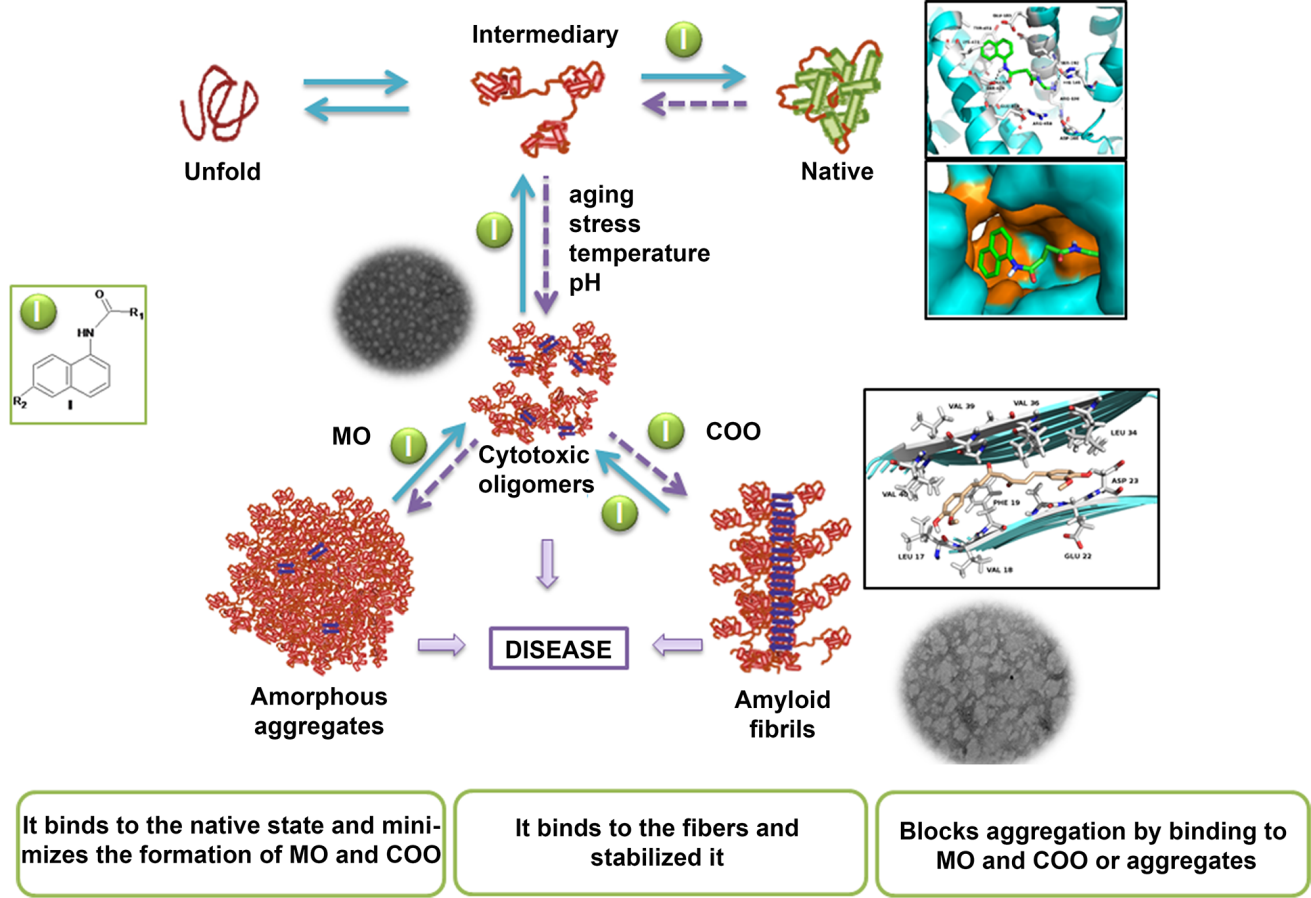
Proposal mechanism of chaperone on protein aggregation/disaggregation. Novel chemical chaperones family can regulate fiber formation processes by binding to the native state, minimizing the formation of amorphous aggregates, as well as cytotoxic oligomers.

In this article, we show how by selecting compounds based on the two states of the amyloid protein (native and amyloid pharmacophore structure) we managed to obtain chaperones that bind to the native state, to the cytotoxic oligomers and to the amyloid fibrils (Figs 6, 9 and 14 and Table 1). The novel chemical chaperones can regulate fibril formation processes by binding to the native state, minimizing the formation of amorphous aggregates, as well as cytotoxic oligomers. It is worth mentioning that these compounds also adhere to the fibrils and stabilize them. Chaperone **F** in particular was found to accelerate the formation of hIAPP_20–29_ fibrils by binding to cytotoxic oligomers (Fig 7). In this article we demonstrate the physiopathological conditions in which a set of chaperones can be used. We observed that in acute processes, with abundance of cytotoxic oligomers, chaperone **F** is the most suitable for arresting the former, forming fibers and thus preventing apoptosis caused by membrane pores. For chronic processes, chaperones **A**, **B**, **C** and **D** were found to be apposite for stabilizing the monomer and reducing the formation of fibres (Table 1, Figs 5–14). This is extremely relevant as it allows for a close examination of the fiber-formation phenomena and, potentially, to control cytotoxicity. If taken to the next stage—assaying the dynamics of these chaperones in living organisms in order to learn their effects within cognitive and metabolic processes—this could lead to the development of therapeutic agents derived from this chaperone family that could help control the devastating effects of conformational diseases. Finally, the findings reported in this article are useful for encouraging research in reverse engineering, as well as building increasingly effective chemical libraries for the treatment of conformational diseases.

## Materials and Methods

MilliQ water (18.2 MΩ resistance at 25°C) for the preparation of all working solutions is used. Glycine, monobasic sodium phosphate, dibasic sodium phosphate, sodium chloride, dimethyl sulphoxide (DMSO) and 3- (4,5-dimethyl-2-thiazoyl) -2,5-diphenyltetrazolium bromide (MTT) from Sigma-Aldrich were used. All buffers were freshly prepared before every aggregation experiment. Thioflavin (ThT) was purchased from Sigma-Aldrich. Bovine serum albumin (BSA, fraction V) was purchased from Roche and stored at 4°C. Stock solution of BSA (700 μmol/L) was freshly prepared by dissolving it in glycine buffer (50 mmol/L, pH 3; NaCl 100 mmol/L), and then passed through acrodisc syringe filters with 0.45 μm pores (Sigma Aldrich). Accurate protein concentration was determined by using BSA molar extinction coefficient at 280 nm (43 824 Lmol−1 cm^−1^).[12] hIAPP fragment 20–29 (hIAPP_20–29_) was synthesized according to the procedures described in the literature.[47–49] This peptide was purified by semi preparative reverse high performance liquid chromatography (SP-HPLC) and its purity was greater than 95%. The expected molecular mass (MM: 1029 Da) was confirmed by mass spectrometry (ESI-MS). Chemical chaperones (A, B, D, E, F and G) were gently provided from Neurosciences Center of Cuba [27,28]. Solutions of each chaperone (25 mmol/L) were prepared in DMSO and stored at -20°C. Naproxen and curcumin, used as reference substances, were dissolved in DMSO and ethanol absolute (25 mmol/L), and then stored at -20°C y a -70°C, respectively. Cerebellar granule cells (CGC) cultures (8 DIV), which were prepared as previously described [44–46], were donated by Dr. J. Moran Institute of Cellular Physiology, UNAM, Mexico. Kinetic assays were monitored by fluorescence spectroscopy and microscopy techniques. Fluorescence measurements were performed on Cary Eclipse fluorescence spectrophotometer (methacrylate cell) and Infinite M1000, TECAN (96-well plates, polystyrene, Costar 3615, Special Optics Plate), equipped with temperature control. Transmission electron microscopy (TEM, JEM-1010, JEOL, 80 kV, magnifications between 1 000 and 120 000) and atomic force microscopy (AFM, Veeco Bioscope, Digital Instruments, mounted on an inverted microscope) were performed.

### Experimental Procedure

#### Kinetic assays of fiber formation. Fluorescence measurements

Aggregation kinetics were followed at 65°C at different times.

#### BSA

Stock solution of BSA (700 μmol/L) was diluted using glycine buffer (50 mmol/L, pH 3; NaCl 100 mmol/L) to yield a final BSA concentration of 100 μmol/L as described by Bhattacharya, et al.[12]. Aliquots of 250 μL of mixtures of BSA:chemical chaperones (**A**, **B**, **C**, **D**, **E**, **F** and **G**) (1:1) were prepared and mixed with 1 μL of ThT (final concentration 20 μmol/L) and incubated during different times at 65°C. The samples were cooled in order to stop the aggregation process as described by Vetry et al [9].These aliquots were deposited into the wells of a 96-well plate, by sextuplicate. For detecting the fiber formation, ThT fluorescence intensity was measured in an Infinite M1000 spectrophotometer, at excitation and emission wavelengths of 450 and 482 nm, respectively. Signals were corrected with the background signal (ThT blank).

#### Fiber Disaggregation Experiments

BSA was pre-aggregated for 24 h at 75°C before incubation with chemical chaperones **A**, **B**, **C**, **D**, and **E** at 1:1 molar ratio, in buffer Tris (pH = 7.4, 20 mmol/L). The samples were applied (6 μL) to 400-mesh copper grids coated with carbon/collodion film, and left for 3 minutes. Excess solution on the grids was absorbed with filter paper, grids were stained with 3μL of 1% filtered uranyl acetate for 5 min, and staining solution was absorbed with filter paper again. After air drying, grids were examined with an electron microscope JEM-1010, JEOL, 80 kV, magnifications between 1 000 and 120 000.

#### hIAPP_20–29_

Stock solution of hIAPP_20–29_ (5.8 mmol/L) was diluted using PBS buffer (100 mmol/L, pH 7,4 with sodium chloride 100 mmol/L) to prepare experiments at a final hIAPP_20–29_ concentration of 100 μmol/L. Experiments were carried out in the methacrylate cells (4.5 mL, 10 mm path length, Sigma Aldrich) in the presence of 1 μL of Th-T (20 mmol/L). Aliquots of 1.5 mL of mixtures of hIAPP_20–29_:chemical chaperones (25 mmol/L, **A**, **B**, **C**, **D**, **E**, **F** and **G**) (1:1) were incubated at 25°C with stirring. IF values were recorded continuously to λemis = 482 nm, exciting at 450 nm with a bandwidth of 5 nm in a Cary Eclipse spectrofluorometer. Signals were corrected with the background signal (ThT blank). The tlag was obtsined by fitting the data to a sigmoidal function (Botzmann function), using Origin Software.

#### IC50

Stock solution of BSA (700 μmol/L) was mixed with chaperones **A**, **B**, **C**, **D** and **E** solutions (25 mmol/L) to prepare mixtures of BSA (300 μmol/L): Chaperones (0, 15, 150, 300, 750 and 1500 μmol/L) and ThT (24 μmol/L) to reach a final volume of 250 μL into the wells of a 96-well plate. The plate was read in an Infinite M1000 spectrofluorometer every 20 min at 42°C. All the ThT fluorescence experiments were replicated six times. For the calculation of IC50, the maximum velocities were computed and plotted against the Log Chaperone concentrations.

#### Microscopy measurements. Transmission Electron Microscopy (TEM)

The samples BSA:chaperones A, B, C, D, E, F, and G (1:1) and BSA from kinetic assays at 65°C were diluted 1/10 or 1/20 in glycine buffer (50 mmol/L, pH 3; NaCl 100 mmol/L) and applied (6 μL) to 400-mesh copper grids coated with carbon/collodion film, and left for 3 minutes. Excess solution on the grids was absorbed with filter paper, grids were stained with 3 μL of 1% filtered uranyl acetate for 5 min, and staining solution was absorbed with filter paper again. After air drying, grids were examined with an electron microscope JEM-1010, JEOL, 80 kV, magnifications between 1 000 and 120 000.

#### Atomic force microscopy (AFM)

The samples BSA:chaperones **A**, **B**, **C**, **D**, **E**, **F**, and **G** (1:1) and BSA from kinetic assays at 65°C were diluted with milli-Q water in a 1:9 (v/v). 100 μL of the diluted sample were deposited on a mica and dried under vacuum over moisture-free silica gel. After drying micas, 500 μL of milli-Q water was added and allowed to stand for 5 min before removing the excess of water using filter paper. The micas were dried again for 24 hours and deposited over glass slides used in light microscopy. The samples were observed with an atomic force microscope (Bioscope, Digital Instruments, Santa Barbara CA, USA, equipped with a Nanoscope IIIa controller) working in contact mode with a silicon nitride probe (DNP, Veeco, USA). Microscope works on an inverted Diaphot 200 (Nikon) microscope with a 100 microns scanner and silicon nitride tips with 50 nm radius of curvature (DNP–Veeco). Sweep speeds used between 1969–1285 Hz, 10 nN force and 0.5 arbitrary units of gain.

#### Cell viability and apoptosis of cerebellar granule cells (CGC). Exposure of CGC cultures to hIAPP_20–29_, as cytotoxic agent

Solutions of hIAPP_20–29_ in PBS buffer (pH 7.4, 0.1 mol/L, 0.1 mol/L NaCl) were prepared. Monomeric and aggregated peptide concentration were 5.8 mmol/L and 32.6 mmol/L, in DMSO, respectively. Viability tests were performed according to the procedures reported by Moran et al. [50]. In particular, in this experiment, the viability of CGC cultures (8 DIV) exposed to hIAPP_20–29_, in its monomeric and aggregated form, was evaluated. CGC cultures were exposed or not to chaperones **B** and **D** at different molar ratios hIAPP_20–29_: chaperones (1:0.01, 1:0.3 or 1:1) and **H** as reference. In brief, in all samples of CGC, half the volume of medium is removed to add, independently, hIAPP_20–29_ (monomeric or aggregated): chaperone at different molar ratios. Next, the enriched cell culture was reintroduced and again incubated at 37°C. After 4 h, the apoptosis was estimated by measuring caspase-3 activity (fluorogenic method according to Moran et al.) or after 20 h, the viability levels were assessed by the MTT test.[51]

#### Exposure of CGC cultures to low potassium medium

CGC cultures (8DIV) containing KCl (25 mmol/L, K25) were transferred to the same medium containing KCl (5 mmol/L, K5). The chaperones **B**, **D** and curcumin as reference (25 μmol/L) were independently added to cultures, at the initial time (Group I) or after 4 h (Group II) of the induction of the pro-apoptotic stimulus (K5) and incubated 4 h additional. Also, negative (K5) and positive (K25) controls were carried out. Apoptosis was estimated by measuring caspase-3 activity (fluorogenic method according to Moran et al.) [51].

### Molecular docking

The crystallographic structure of the BSA was downloaded from Protein Data Bank (PDB) (3V03 code), with a resolution of 2.7 Å. The·3D fibril structure of the Aβ_17-42_ amyloid peptide, as a NMR image, was downloaded from the PDB (2BEG code) with 10 configurations [31].The structures of the chemical chaperones were refined using *Avogadro* software and converted into pdbqt format with *Autodock Tools*, considering all ligand bonds as flexible. These compounds were docked with both proteins using *AutoDock Vina*, an interactive molecular graphics program used to understand the drug-protein interaction. *Vina* uses a scoring function, which can be seen as an attempt to approximate the standard chemical potentials of the system, in order to search for the optimum binding site of small molecules into the protein. For these simulations all amino acids of tested protein were considered in rigid position. Folders with the data for the calculation were compiled each file of which contained the BSA and Aβ_17-42_ configurations (pdbqt) and the corresponding ligand configuration (pdbqt). During docking, a maximum of 10 conformers was considered for each molecule. To recognize the binding sites in BSA or Aβ_17-42_ a blind docking was carried out. The grid size set to 108, 100, and 100 along X, Y and Z axes with 1.00 Å grid spacing. Thus, each ligand can move freely across the surface of the BSA or Aβ_17-42_. The center of the grid was set to 38.00, 25.00, and 36.00 Å. The visualization and determination of the interaction zones between the amino acids and all tested compounds were carried out using the molecular graphic program (Chimera). All calculations were performed on a cluster of 10 computers (30 CPU) with Linux as operating system.

#### Statistical analysis

STATISTICA software, version 8, was used and the significance level was set at 0.05. Experimental data from kinetic assays were processed using OriginPro 8.5 program.

## Supporting Information

S1 Table Energy of the binding complex (chaperones-BSA) and interaction sites of BSA-chaperonines obtained from molecular docking using AutoDockingVina. (DOC)
