# Supplementary material for: Drug Development in Conformational Diseases: A Novel Family of Chemical Chaperones that Bind and Stabilise Several Polymorphic Amyloid Structures
Source: PLoS One. 2015 Sep 1;10(9):e0135292. doi: 10.1371/journal.pone.0135292 (PMC4556714; doi:10.1371/journal.pone.0135292)
Supplement: S1 Table — (DOC) [file pone.0135292.s001.doc]

| **Tested Chaperonines** | **Groups** | **ΔG (Kcal/mol)** | **Interaction sites of BSA with chaperonines** |
| --- | --- | --- | --- |
| F | 3G | -9.5 | L112-D118 L122 E125 F133 K136 Y137 E140 I141 Y160 R185 V187 |
| E | 2G | -8.4 | L115-D118 L122 E125 F133 K136 Y137 E140 I141 R145 Y160 I181 R185 |
| D | 2G | -8.3 | L115-P117 L122 E125 K136 Y137 E140 I141 Y160 I181 M184 R185 V187 |
| G | 1G | -8.2 | L115-D118 L122 E125 F133 K136 Y137 E140 Y160 I181 E182 M184 R185 |
| A | 1G | -7.8 | L115-D118 T121 L122 E125 F133 K136 Y137 E140 Y160 |
| B | 3G | -7.8 | L115-D118 T121 L122 E125 F133 K136 Y137 E140 I141 Y160 I181 R185 |
| C | 4G | -7.4 | L115-P117 L122 F133 K136 Y137 E140 I141 Y160 I181 R185 |

S1 Table. Energy of the binding complex (chaperones-BSA) and interaction sites of BSA-chaperonines obtained from molecular docking using AutoDockingVina.
